# Exclusive Cutaneous and Subcutaneous Sarcoidal Granulomatous Inflammation due to Immune Checkpoint Inhibitors: Report of Two Cases with Unusual Manifestations and Review of the Literature

**DOI:** 10.1155/2019/6702870

**Published:** 2019-07-09

**Authors:** Narciss Mobini, Rummit Dhillon, Jason Dickey, Jordan Spoon, Kaviyon Sadrolashrafi

**Affiliations:** ^1^University of Nevada, School of Medicine, Reno and Las Vegas, Nevada, USA; ^2^Associated Pathologists Chartered, Las Vegas, Nevada, USA; ^3^College of Osteopathic Medicine, Touro University, Henderson, Nevada, USA; ^4^Thomas Dermatology, Las Vegas, Nevada, USA; ^5^University of California, Los Angeles (UCLA), California, USA

## Abstract

Recent emergence of immune checkpoint inhibitors (ICIs) has revolutionized the treatment of cancers and produced prolonged response by boosting the immune system against tumor cells. The primary target antigens are cytotoxic T-lymphocyte-associated antigen-4 (CTLA-4), a downregulator of T-cell activation, and programmed cell death-1 receptor (PD-1), a regulator of T-cell proliferation. This enhanced immune response can induce autoimmune adverse effects in many organs. Although skin toxicities are the most common, sarcoidal inflammation with exclusive cutaneous involvement is a rare occurrence with only 6 cases reported to date. We report 2 cases with unusual features. One patient is a female who was treated for metastatic renal cell carcinoma with combination of ipilimumab (anti-CTLA-4) and nivolumab (anti-PD-1). She developed deep nodules showing sarcoidal dermatitis and panniculitis on histopathologic exam. The second patient is a male with melanoma of eyelid conjunctiva who was treated prophylactically with ipilimumab. He presented with papules/plaques confined to black tattoos, where the biopsy revealed sarcoidal dermatitis. By a comprehensive literature review, we intend to raise awareness about this potential skin side effect in the growing number of patients receiving targeted immunotherapies. It is crucial to have a high index of suspicion and perform timely biopsies to implement appropriate management strategies.

## 1. Introduction

Despite their tremendous success in treatment of cancer, ICIs are capable of inducing a variety of immune-related adverse events in many organ systems. Skin is reported to be the most common organ affected among other organs such as gastrointestinal, hepatic, endocrine, and renal [1]. The incidence of dermatologic toxicities from ipilimumab (anti-CTLA-4) in metastatic melanoma patients ranges from 49% to 68%, compared to 24% risk of toxicity used for other cancers such as urothelial carcinoma, pancreatic adenocarcinoma, renal cell carcinoma, and non-small cell lung carcinoma [2]. The most common cutaneous side effects related to ipilimumab are pruritus, morbilliform rash, maculopapular eruptions resembling a dermal hypersensitivity reaction, vitiligo, and lichenoid reactions [3]. With anti-PD-1 drugs there may be a 34-39% chance of such adverse cutaneous reactions [1, 4]. Other less common cutaneous toxicities collectively include lichenoid mucositis (tongue, buccal, gingiva, and lips), exacerbation of psoriasis, immunobullous lesions, erythema multiforme, exfoliative dermatitis, prurigo nodularis, pyoderma gangrenosum-like ulceration, Sweet syndrome, DRESS syndrome, and toxic epidermal necrolysis [5–7]. Sarcoidal-type granulomatous dermatitis, a rare occurrence, was first introduced by Eckert et al. in 2009 as an adverse side effect of ipilimumab for metastatic melanoma [8]. In addition to ICI, it is noteworthy that sarcoidal lesions can also appear during treatments with kinase inhibitors such as BRAF/MEK inhibitors [6]. ICI-induced cutaneous sarcoidal reactions have been reported in only six patients in the literature to date [2, 9–12]. We present two new cases of such reactions with unique and exclusively skin manifestations following immune checkpoint inhibitors.

## 2. Case Reports

### 2.1. Case # 1

**Figure 1:**
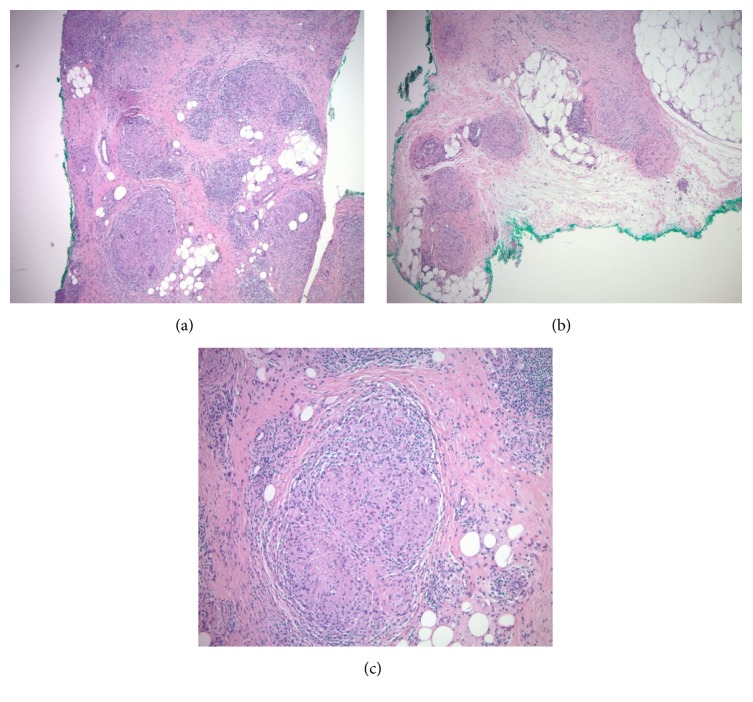
(a), (b), (c). Deep dermal and mainly subcutaneous sarcoidal granulomata characterized by multinodular infiltrate of mono- and multinucleated epithelioid histiocytes with some lymphocytes (hematoxylin-eosin stain, magnifications 10 X, 10X, 20X, respectively).

A 49-year-old female was referred by her oncologist for evaluation of deep nodules on the left elbow and left forearm for 2 months. She had a history of renal cell carcinoma, clear cell type, and was treated by radical nephrectomy one year prior to her visit. The tumor was reported to be limited to the kidney cortex with no lymphovascular invasion or regional lymph node metastasis (TNM:T2b, NX). Seven months later, the patient developed metastatic lung lesions. She was then treated with nivolumab (opdivo) and ipilimumab (yervoy). The patient started to develop slowly enlarging subcutaneous lesions on her left forearm and elbow one month after the first round of therapy. The patient has a family history of Fragile X syndrome in two of her three sisters and in two brothers, one of whom is also blind. Her parents and children are healthy. On physical examination, there were large nontender firm subcutaneous nodules and plaques on her left forearm and elbow, which were more palpable than visible. A skin biopsy was performed that revealed sarcoidal-type granulomatous inflammation in the dermis and subcutaneous tissue (Figures 1(a), 1(b), and 1(c)). Examination with polarized light failed to reveal foreign material. Special stains for fungi (PAS/periodic acid-Schiff) and atypical mycobacteria (AFB and Fite) were negative. In addition, due to the patient's immunocompromised state, appropriate cultures from the affected skin were also obtained that yielded negative results. The sarcoidal dermatitis and panniculitis was therefore believed to be secondary to combination therapy with opdivo and yervoy. Upon consultation with the treating oncologist, the checkpoint inhibitor therapy was decided to be discontinued after the third round. Systemic workup failed to reveal sarcoidal lesions elsewhere in her body. On the subsequent follow-up visit in three weeks, the patient reported that the lesions were decreasing in size and firmness. She started a new regimen at this time.

### 2.2. Case # 2

**Figure 2:**
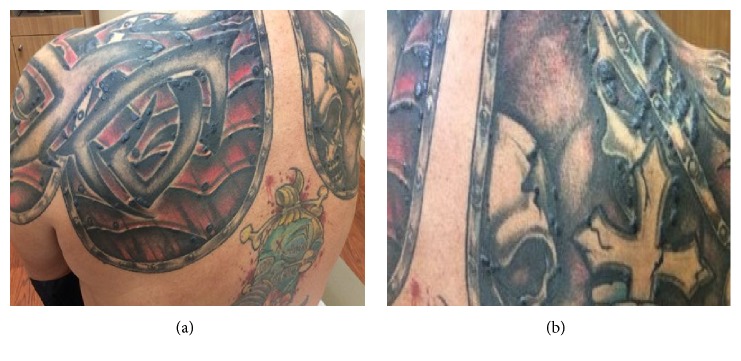
(a), (b). Papulonodular lesions within the black tattoos in patient 2.

**Figure 3:**
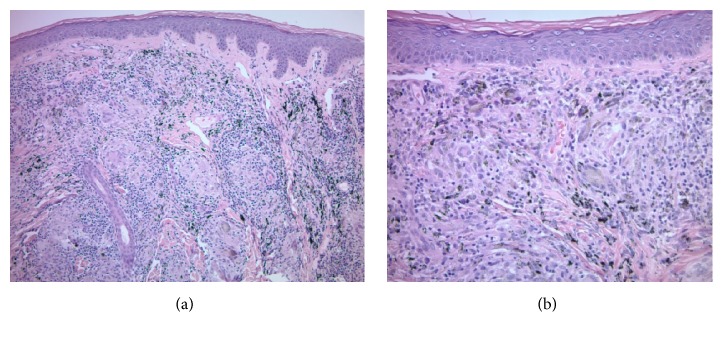
(a), (b). Sarcoidal-type granulomatous inflammation confined to the areas harboring black tattoo pigment (hematoxylin-eosin stain, magnifications 10 X, 20X, respectively).

A 58-year-old male presented with lesions occurring only within his black tattooed skin on the chest, shoulders, back, left forearm, and right thigh for the past 3 months. The lesions were tender (only upon pressure), with no itching or pain. All tattoos were present for more than 5 years. The patient has a medical history of hypertension and eczema, with a family history of colon cancer in both parents. He was diagnosed with malignant melanoma on the left eyelid conjunctiva 8 months earlier, measuring 1.8 mm with ulceration (TNM stage: pTN2b). Sentinel lymph node from the left preauricular region was negative. Melanoma was treated with Mohs surgery and wide local excision. Metastatic workup was negative. He was later started on four rounds of adjuvant ipilimumab prophylaxis. The rash appeared after the first month of treatment. On physical examination, there were erythematous, scaly tender papules, plaques, and nodules, only confined to the black tattooed areas on his chest, shoulders, upper back, left forearm, and right thigh. The red, yellow, and green tattoos were completely uninvolved (Figures 2(a) and 2(b)). With the clinical diagnosis of possible allergic reaction, he was initially treated with oral prednisone 10 mg/day and 0.1% triamcinolone cream for two weeks with some improvement; however, the rash was persistent. Treatment was switched to topical clobetasol cream and he was given an intralesional triamcinolone acetonide (kenalog) injection to an area on the right upper arm. In his 4-week follow-up visit, due to the lack of significant clinical improvement, a punch biopsy from the left upper arm was performed that revealed sarcoidal-type granulomatous inflammation, associated with only the black tattoo areas (Figures 3(a) and 3(b)). Since the tattoos were present for many years prior to this occurrence with no such reactions, we concluded that the sarcoid reaction was secondary to his ICI therapy. The results were communicated to his oncologist and the ICI treatment was decided to be stopped. A systemic workup failed to reveal lesions elsewhere in his body. In subsequent follow-up visits, the lesions started to improve without further treatment. He is currently being seen by his oncologist at regular intervals, who will continue to monitor the patient for internal disease.

## 3. Discussion

Immune-related adverse events are well-recognized consequences of immunotherapies. Sarcoidal lesions can appear during treatments with both kinase inhibitors such as BRAF/MEK inhibitors and immune checkpoint inhibitors [6, 12]. During ICI therapies, sarcoidal reactions most commonly involve hilar, mediastinal, or thoracic lymph nodes and also pulmonary parenchyma. It is not certain if the development of sarcoidal lesions carries a better prognosis in patients receiving ICI treatments. In 71% of patients with sarcoidal reactions due to ICIs, the malignancy showed either a partial clinical response, remained stable, or went into remission. In 29% of reports, the malignancy progressed. More than 90% of sarcoidal lesions resolved or improved, irrespective of the medical intervention [13]. In 38-49% of the patients, immunotherapy was discontinued, 44-57% of patients were given systemic steroids for their lesions, and local steroid treatment was used in 8 to 24% of reported cases [4, 13]. Both of our patients showed only cutaneous and/or subcutaneous involvement, with no systemic involvement and there was no prior history of sarcoidosis in either one. Of note, the therapies were given for metastatic renal cell carcinoma (Pt1) and as adjuvant therapy and prophylactically for a conjunctival melanoma (Pt2). Sarcoidal lesions are mostly reported in the setting of treatment for metastatic melanomas. To our knowledge, we report the first case of sarcoidal granulomatous inflammation following ICI therapy that has remained confined within the black tattoo on the skin. Only one case of tattoo sarcoid has been reported, but additional skin areas and the hilar lymph nodes were also involved [14]. One interesting note is that papulonodular reactions in black tattoos are strong markers of sarcoidosis. The “Rush phenomenon” begins with a recent tattoo triggering a local papulonodular reaction. It is characterized by a concomitant reaction in many other black tattoos on the individual and has proven to be a sarcoidal reaction in the majority of cases. Sarcoidosis is estimated to be increased 500-fold in papulonodular reactions compared to the prevalence in the general population [15]. In our patient, the tattoos were present for more than 5 years with no history of any reactions. Therefore, we deduce that the ICI therapy must be the main culprit in producing this manifestation.

Table 1 summarizes all the previously reported cases of sarcoid/sarcoid-like reactions from ICI therapy so far that clinically involved the skin, with or without other organ involvement [13, 14, 16–32].

In summary, of 36 total cases (including our current two cases) reported to date, 24/36 or 67% were female and 12/36 or 33% were male. Exclusive cutaneous/subcutaneous involvement was reported in 8/36 or 22%, including our present cases. The most common site of skin involvement was upper and lower extremities. Other locations included face, scalp, chest, and trunk. Two cases showed tattoo involvement where in one, the sarcoid reaction was only confined to black tattoo (current report). In addition, localization to dermal scars was seen in two patients. Lymph nodes were the most common extracutaneous organ involved in 15/36 or 45% of cases, followed by pulmonary parenchyma in 11/36 or 30%. Ipilimumab was the culprit in 11/36 or 31%, nivolumab and pembrolizumab, each in 8/36 or 22%, and combination therapy was reported in 9/36 or 25%. The most common type of underlying cancer was melanoma in 30/36 or 83%, which is consistent with the previously published research on ICI-induced sarcoidal reactions reported to occur in more than %75 of the patients under melanoma treatment [13]. Melanomas are highly immunogenic and the neoantigen environment in these cells has a tremendous impact on antitumor activity of cytotoxic T cells and response to ICI. Enhanced destroying of melanoma cells induced by ICI therapy exposes additional neoantigens presenting by antigen presenting cells that promote Th1 response and release of cytokines that promote the development of granulomatous/sarcoidal lesions in ICI therapy. The pathogenesis of sarcoidal granulomas is complex and involves the interaction of mononocytes/macrophages and CD4 + Th1 cells. In response to antigens and possibly neoantigens secondary to destruction of melanoma in ICI therapy, macrophages produce TNF-alpha and interleukins that recruit CD4+ Th cells [33]. Cytokines that enhance Th1 differentiation are found to be upregulated in sarcoidosis where they secret IFN-gamma and IL-17 and organize granulomatous structure by promoting the maturation of epithelioid histiocytes and multinucleated giant cells. Sarcoidosis seen during ICI therapy supports a hyperactive immune response. Recent reports also highlight the possible role of Th17 cells in the pathogenesis of sarcoid, specifically a subset of CD4+T cells that produce IFN-gamma and IL-17 [34, 35]. Although the development of sarcoidal lesions in immunotherapies may represent a favorable sign of potential therapeutic response, it is not yet completely elucidated and requires further studies in larger scales for clearer guidelines in the clinical management of these patients.

## 4. Conclusion

Immune checkpoint targeted agents can induce nonspecific enhanced immune response and overstimulation of inflammatory pathways, leading to a spectrum of autoimmune side effects. Among these, sarcoidosis or sarcoid-like lesions are reported with the majority of cases, presenting with lymph node and pulmonary involvement, and less frequently skin and other organs. By reporting two new cases of exclusively cutaneous/subcutaneous sarcoid secondary to ipilimumab and nivolumab immunotherapies (so far there are a total of 8 cases in the literature) and a thorough review of existing published data, we intend to raise awareness of this potential adverse effect. To our knowledge, we report the first case of sarcoidal granulomatous dermatitis confined solely to black tattoo areas with no systemic involvement. In light of increased utilization of successful ICI therapies today, clinicians should have a high index of suspicion and perform timely biopsies of any newly developing, unusual, or persistent cutaneous lesions in the course of the treatment to avoid misinterpreting sarcoid reactions as progressive or recurrent disease and implement proper management strategies.

## Figures and Tables

**Table 1:** Sarcoidal-type granulomatous reaction due to immune checkpoint inhibitors (anti-CTLA-4 and anti PD-1 antibodies), affecting skin, with and without other organ systems.

	Case	Age	Sex	Primary disease	Clinical manifestation	ICI therapy
1	Suozzi, 2016 [9]	60	F	Lung Adenocarcinoma	Cutaneous only	Ipilimumab & Nivolumab

2	Ogawa, 2018 [10]	63	F	Lung Adenocarcinoma	Cutaneous only	Nivolumab

3	Birnbaum, 2017 [11]	56	F	Melanoma	Cutaneous only	Nivolumab

4	Dimitriou, 2018 [12]	72	M	Melanoma	Cutaneous only	Pembrolizumab

5	Tetzlaff, 2017 [2]	57	F	Ovarian Cancer	Subcutaneous only (bilateral lower extremities, forearms)	Ipilimumab & Nivolumab

6	Tetzlaff, 2017 [2]	39	F	Melanoma	Subcutaneous only (bilateral lower extremities & buttocks)	Ipilimumab & Nivolumab

7	Current Report, 2019	49	F	Renal Cell Carcinoma	Cutaneous only (left forearm and elbow)	Ipilimumab & Nivolumab

8	Current Report, 2019	58	M	Melanoma	Cutaneous only (black tattoo on chest, shoulders, back, forearm, thigh)	Ipilimumab

9	Kim, 2016 [14]	52	M	Urothelial Carcinoma	Cutaneous (tattoo & face), Hilar Lymph Nodes (LN)	Ipilimumab & Nivolumab

10	Cotliar, 2016 [16]	72	F	Hodgkin Lymphoma	Subcutaneous (bilateral arms), Pulmonary, Hilar LN, Bone	Pembrolizumab

11	Tetzlaff, 2018 [13]	44	F	Melanoma	Subcutaneous (peripatellar), Hilar LN	Ipilimumab

12	Tetzlaff, 2018 [13]	79	M	Melanoma	Cutaneous (bilateral forearms, elbows, hands), Hilar LN	Pembrolizumab & Nivolumab

13	Le Burel, 2017 [17]	71	F	Melanoma	Cutaneous, Pulmonary Parenchyma	Nivolumab

14	Lomax, 2017 [18]	75	F	Melanoma	Cutaneous, Pulmonary	Nivolumab

15	Oommen, 2017 [19]	54	M	Melanoma	Cutaneous, Pulmonary	Ipilimumab

16	Reddy, 2017 [20]	57	F	Melanoma	Cutaneous, Pulmonary	Ipilimumab

17	Reddy, 2017 [20]	55	F	Melanoma	Cutaneous, Pulmonary	Ipilimumab & Nivolumab

18	Toumeh, 2016 [21]	26	F	Melanoma	Cutaneous, Pulmonary	Ipilimumab

19	Yatim, 2018 [22]	72	F	Melanoma	Subcutaneous (left flank, right leg, scar), Pulmonary, hilar LN, Eye	Pembrolizumab

20	Firwana, 2017 [23]	37	F	Melanoma	Cutaneous (right forearm, bilateral shins), Hilar LN & other LN	Ipilimumab & Pembrolizumab

21	Firwana, 2017 [23]	57	F	Melanoma	Cutaneous (bilateral lower extremities), Hilar LN	Ipilimumab

22	Danlos, 2016 [24]	57	M	Melanoma	Cutaneous (lip, scar), Hilar and Mediastinal LN	Nivolumab

23	Martinez, 2016 [25]	46	F	Melanoma	Cutaneous, Pulmonary	Ipilimumab

24	Tissot, 2013 [26]	57	M	Melanoma	Cutaneous (elbow, arm), Pulmonary, Hilar LN	Ipilimumab

25	Eckert, 2009 [8]	67	F	Melanoma	Cutaneous, Pulmonary	Ipilimumab

26	Reule, 2013 [27]	55	M	Melanoma	Cutaneous (finger, forearm, knee), Pulmonary, Hilar LN	Ipilimumab

27	Lomax, 2017 [18]	57	F	Melanoma	Subcutaneous & Cutaneous (elbows, wrists, thighs), Pulmonary, Hilar LN	Pembrolizumab

28	McKenna, 2018 [28]	69	F	Melanoma	Cutaneous, Pulmonary	Nivolumab

29	Jespersen, 2018 [29]	57	F	Melanoma	Cutaneous, Pulmonary, Bone	Pembrolizumab

30	Dimitriou, 2018 [12]	65	M	Melanoma	Cutaneous (elbow), Hilar LN	Pembrolizumab

31	Dimitriou, 2018 [12]	57	M	Melanoma	Cutaneous (scar), Hilar LN, Thyroid	Ipilimumab & Nivolumab

32	Burillo-Martinez, 2017 [30]	69	F	Melanoma	Subcutaneous (forearms, legs, blue nevus), Hilar LN	Pembrolizumab

33	Paolini, 2018 [31]	56	F	Non-small Cell Lung Cancer	Cutaneous, Pulmonary	Nivolumab

34	Wilgenhof, 2012 [32]	37	M	Melanoma	Cutaneous, Pulmonary, Lymph Nodes, Spleen	Ipilimumab

35	Lomax, 2017 [18]	44	M	Melanoma	Cutaneous (elbow and occipital region), Pulmonary, Hilar and Mediastinal LN, Colitis	Nivolumab

36	Lomax, 2017 [18]	65	F	Melanoma	Cutaneous (face, knees), Thyroiditis, Pulmonary, Hilar LN	Pembrolizumab
